# Systemic Lipidomics Identifies Altered Ceramide–Phospholipid Balance Associated With Periodontitis Severity: A Cross‐Sectional Study With 12‐Year Follow‐Up

**DOI:** 10.1111/jcpe.70182

**Published:** 2026-08-02

**Authors:** Minja Tuomivaara, Anne‐Mari Halme, Muhammed Manzoor, Elina Salasuo, Susanna Paju, Päivi Mäntylä, Kåre Buhlin, Laura Lahdentausta‐Suomalainen, Reijo Laaksonen, Juha Sinisalo, Pirkko Pussinen, Aino Salminen

**Affiliations:** ^1^ School of Medicine, Institute of Dentistry University of Eastern Finland Kuopio Finland; ^2^ Oral and Maxillofacial Diseases University of Helsinki and Helsinki University Hospital Helsinki Finland; ^3^ Institute of Odontology Karolinska Institutet Huddinge Sweden; ^4^ Zora Biosciences Oy Espoo Finland; ^5^ Heart and Lung Center Helsinki University Hospital, and University of Helsinki Helsinki Finland

**Keywords:** Biomarkers, cardiometabolic risk, lipidomics, periodontal diseases, sphingolipids, systemic inflammation

## Abstract

**Aim:**

To investigate whether periodontitis stage and grade are associated with circulating active lipid mediators and whether baseline lipid species predict periodontitis progression.

**Materials and Methods:**

The study included 473 Finnish participants from the Parogene cohort undergoing coronary angiography, with 12‐year follow‐up data available for 117 individuals. Periodontal status was assessed clinically and radiographically. Plasma ceramides (Cer) and phosphocholines (PC) were quantified by LC‐MS/MS, and cardiovascular risk scores (CERT1 and CERT2) were calculated.

**Results:**

Several Cer:Cer and Cer:PC ratios increased with periodontitis stage, grade and edentulousness, whereas most PC species decreased. Cer(d18:1/16:0)/PC(16:0/22:5), Cer(d18:1/18:0)/Cer(d18:1/24:0), Cer(d18:1/18:0)/PC(16:0/22:5) and Cer(d18:1/24:1)/PC(16:0/22:5) were associated with all periodontal measures. CERT2 was independently associated with both stage and grade. In cross‐sectional analyses, periodontitis was associated with multiple lipid species both directly and indirectly through systemic inflammation. In longitudinal analyses, baseline lipid levels did not predict periodontitis progression.

**Conclusion:**

Periodontitis severity was found to be associated with systemic ceramide and phospholipid profiles, particularly Cer:PC ratios, whereas baseline lipid levels were not associated with subsequent disease progression. These findings suggest that systemic inflammation substantially mediates the relationship between periodontitis and circulating lipid species, linking periodontitis with broader disturbances in lipidomic homeostasis.

## 1 Introduction

Periodontitis has been increasingly linked to a range of cardiometabolic diseases, including cardiovascular diseases (Larvin et al. 2021), metabolic syndrome (Kinnunen et al. 2025) and type 2 diabetes mellitus (Lalla and Papapanou 2011). These associations are thought to be mediated by systemic inflammation, immune system dysregulation and the dissemination of microbial products from periodontal pockets into the circulation or down the gastrointestinal tract (Hajishengallis and Chavakis 2021; Kitamoto and Kamada 2022). Despite these observations, the biological mechanisms underlying these links remain incompletely understood. Metabolomic studies suggest that alterations in lipid metabolism may link periodontitis to systemic diseases, either contributing to or arising from chronic inflammatory processes (Salminen et al. 2024; Basdorf et al. 2024).

Phospholipids (PLs) and sphingolipids, including ceramides (Cer), are essential structural and functional components of cellular membranes. Beyond maintaining membrane integrity, they participate in signalling pathways, modulating inflammatory responses (Huang et al. 2011; Hannun and Obeid 2018; van Meer and de Kroon 2011). Phosphatidylcholines (PCs), the most abundant phospholipid species in mammalian membranes and circulating lipoproteins, play central roles in membrane fluidity, lipid transport and lipoprotein assembly. Alterations in PC composition have been linked to inflammatory responses, with some species promoting oxidative stress and others exhibiting anti‐inflammatory properties (Bochkov et al. 2010; Mizuno et al. 2025).

Ceramides are bioactive sphingolipids consisting of a sphingosine backbone linked to a fatty acid. They regulate key cellular processes such as apoptosis, proliferation, senescence and inflammation (Canals et al. 2018; Hannun and Obeid 2018; Chaurasia et al. 2020). Specific ceramide species have been associated with systemic inflammatory and metabolic alterations. For example, Cer(d18:1/18:0) has been linked to increased inflammatory activity (Havulinna et al. 2016). These findings highlight the importance of studying individual lipid species rather than total lipid classes, as their biological functions may differ substantially.

Given their roles in inflammation and cellular signalling, ceramides and phospholipids are plausible components of systemic responses associated with periodontal disease. Chronic periodontal inflammation may influence systemic lipid metabolism, leading to measurable alterations in circulating lipid profiles (Salminen et al. 2024; Basdorf et al. 2024). Certain ceramide species, as well as oxidised phosphatidylcholines, may also actively modulate inflammatory pathways, suggesting a potential contributory role in amplifying or sustaining the host immune response (Chaurasia et al. 2020). In turn, these lipid alterations may reflect ongoing inflammatory processes and provide insight into disease‐associated systemic changes. Previous studies have demonstrated lipidomic alterations in gingival tissues of patients with periodontitis (Zhou et al. 2022), and lipidomic approaches have identified periodontal bacteria in dental plaque samples (Grundner et al. 2022; Overmyer et al. 2021). However, studies examining circulating lipidomic profiles in relation to periodontitis are limited.

Building on this background, the present study aimed to investigate whether periodontitis is associated with systemic levels of ceramides and phospholipids. In addition, we wanted to examine whether these lipid species are associated with periodontitis progression in a sub‐cohort followed up for 12 years. By integrating detailed periodontal assessments with systemic lipidomic profiling, this study seeks to clarify how the lipidome is altered in periodontitis and whether these changes are related to disease severity and progression.

## 2 Materials and Methods

### 2.1 Study Population

The study population was derived from the COROGENE cohort study consisting of 5294 Finnish patients who underwent coronary angiography in the Helsinki University Central Hospital between 2006 and 2008 (Vaara et al. 2012). From the COROGENE population, a random sample of approximately 10% (*N* = 508) participated in the Parogene sub‐study (permission HUS 426/E5/05), where they underwent a comprehensive clinical oral examination, which included panoramic radiography to estimate alveolar bone loss (Buhlin et al. 2011). The number of periodontal pockets with probing depths (PPD) 4–5 mm and ≥ 6 mm was recorded, as well as the percentage of sites with bleeding on probing (BOP%). All participants signed an informed consent.

A total of 124 (24.4%) participants attended the follow‐up examination with panoramic radiographs taken (permission HUS 152/2016). The examination was performed 12 years (mean 11.9, SD 0.17) later. Those who did not participate in the follow‐up examination were older, had higher concentrations of serum glycoprotein acetyls (GlycA) and fewer teeth than those who participated (Table S1). Lipidome analyses were available for 473 (93.1%) participants at baseline. A total of 117 (24.7%) of those subjects participated in the follow‐up examination.

Periodontitis stage and grade were assessed from the baseline and follow‐up panoramic radiographs (Tonetti et al. 2018; Holtfreter et al. 2024). Based on the tooth with the most severe radiographic bone loss (RBL), staging was performed as follows: stage I, RBL < 15% of the root length; stage II, RBL 15%–33%; stages III‐IV, RBL > 33%. Differentiation between stages III and IV was based on the number of opposing tooth pairs (< 10 or ≥ 10) (Holtfreter et al. 2024). Grade was defined from panoramic radiographs by calculating %RBL/age at the tooth with the most severe RBL without considering modifying factors, diabetes or smoking.

By comparing baseline and follow‐up radiographs, participants were classified as severe (progression of RBL in the worst site > 4 mm during follow‐up or at least one tooth lost due to periodontitis) versus mild progression of periodontitis and those with 0–1 versus > 1 tooth lost.

### 2.2 Clinical Laboratory Analysis

Blood samples were collected at baseline during the angiogram and stored at −80ºC until analysis. Ceramides and phospholipids were quantified using liquid chromatography‐tandem mass spectrometry (LC‐MS/MS). In brief, lipids were separated by liquid chromatography based on their chemical properties. Then the separated compounds were ionised and analysed by the mass spectrometer, which measures the mass‐to‐charge ratio (*m*/*z*) of each ion. In the final step, the selected ions were fragmented and analysed again to verify their identities and quantify them accurately. The lipid species are named according to IUPAC‐IUBMB (International Union of Pure and Applied Chemistry and International Union of Biochemistry and Molecular Biology), and the central reference system LIPID MAPS is used for naming the diverse and structurally complex lipids. The name refers to the lipid structure including detailed fatty chain composition and sphingoid backbone chemistry.

Absolute concentration and calculated concentration ratios were used to calculate each participant's coronary event risk scores CERT1 and CERT2 (Gencer et al. 2022). The CERT1 score is based on six variables: Cer(d18:1/16:0), Cer(d18:1/18:0), Cer(d18:1/24:0) and Cer(d18:1/24:1) and their corresponding ratios to Cer(d18:1/24:0). The CERT2 score extends CERT1 by incorporating specific ceramide/phosphatidylcholine (PC) ratios: Cer(d18:1/24:1)/Cer(d18:1/24:0), Cer(d18:1/16:0)/PC(16:0/22:5), Cer(d18:1/16:0)/PC(14:0/22:6) and PC(16:0/16:0). For both scores, each component was categorised into quartiles and assigned 0–3 points. The total CERT1 and CERT2 scores were calculated as the sum of component scores, ranging from 0 to 12 (Gencer et al. 2022).

Serum GlycA values were available from our previous studies (Salminen et al. 2024; Halme et al. 2025), which were determined at baseline with the high‐throughput NMR metabolomics platform described previously (Würtz et al. 2017).

### 2.3 Statistical Analysis

Statistical differences between frequencies, mean and median values were analysed with Pearson's Chi‐square test, *t*‐test or ANOVA and the Kruskal–Wallis test, respectively. Periodontal stage and grade, as well as edentulousness, were analysed in relation to lipid concentrations by using multivariate linear regression models. Since the associations were not linear, the exposures, that is, oral parameters, were added as bi‐categorical variables. Comparisons were done following periodontal Stages 2–4 versus Stage 1; Grades B and C versus Grade A; complete edentulousness versus presence of teeth; and number of periodontal pockets with PPD 4–5 mm > 5 versus < 5, BOP% Tertiles 2–3 versus Tertile 1. Multivariate logistic regression was used to analyse the associations between lipid species as the exposures and periodontitis progression as the outcome during the follow‐up. Given the relatively high outcome prevalence, odds ratios were converted to approximate risk ratios using the baseline risk of the reference group to improve interpretability. The analyses were adjusted for age, sex, smoking status (never, former, current), BMI and diabetes. Non‐periodontitis subjects were excluded from the analyses concerning periodontitis grade and stage.

We assessed whether the effects of periodontal stage, grade and edentulousness on lipid outcomes were mediated by GlycA. Two models were considered: (i) age and sex, and (ii) age, sex, smoking (never, former, current), BMI and diabetes. Mediation analyses were performed using the R package mediation (v. 4.5.1) with 1000 bootstrap simulations. Average causal mediation effects (ACME), average direct effects (ADE), total effects and proportion mediated were estimated. For lipid‐based cardiovascular risk scores (CERT1 and CERT2), results from Model 2 were presented. Heatmaps were generated in R using *ggplot2* (v.4.0.0). Effect sizes were expressed as the proportion of the total effect mediated, and significance was determined at *p* < 0.05.

## 3 Results

The distribution of periodontitis stage among participants at baseline was 37 (7.8%) stage I; 209 (44.2%) Stage II; 57 (12.1%) Stage III; and 135 (28.5%) Stage IV. Table 1 summarises the characteristics in relation to periodontitis stage. Frequency of smoking, CAD, diabetes, dyslipidaemia and mean age increased with periodontitis severity, whereas dentate and edentulous (*n* = 31 [6.6%]) participants differed significantly only by age. Participants with periodontitis were graded as follows: 42 (9.6%) Grade A; 353 (80.6%) Grade B; and 44 (10.0%) Grade C. Smoking habit, CAD, hypertension and age increased significantly with increasing periodontitis grade (Table S2).

**TABLE 1:** Demographic and clinical characteristics of participants according to periodontitis stage (I–IV) and dentition status.

	Stage I *n* = 37	Stage II *n* = 209	Stage III *n* = 57	Stage IV *n* = 135	*p* ^a^	Edentulous *n* = 31	
	*N* (%)					*N* (%)	*p* ^a^
Females	18 (48.6)	74 (35.4)	19 (33.3)	41 (30.4)	0.223	13 (41.9)	0.394
Smokers^b^	11 (29.7)	96 (45.9)	33 (57.9)	90 (66.7)	**< 0.001**	20 (66.7)	0.132
CAD	20 (54.1)	129 (62.0)	36 (63.2)	112 (83.0)	**< 0.001**	23 (74.2)	0.460
Hypertension	19 (51.4)	134 (64.7)	33 (57.9)	92 (68.1)	0.210	23 (74.2)	0.236
DM I or II	4 (11.1)	41 (19.9)	10 (17.5)	45 (33.6)	**0.004**	11 (35.5)	0.119
Dyslipidaemia	27 (73.0)	160 (77.3)	43 (75.4)	118 (88.1)	**0.041**	26 (83.9)	0.597

Mean (SD)					*p* ^c^	Mean (SD)	*p* ^d^
Age (years)	55.3 (10.9)	62.0 (8.8)	62.7 (8.3)	66.9 (7.1)	**< 0.001**	69.1 (5.7)	**< 0.001**
BMI (kg/$m^{2}$)	27.6 (4.8)	28.2 (5.3)	27.0 (4.7)	27.9 (5.1)	0.462	27.5 (3.4)	0.681
GlycA (μmol/L)	1.15 (0.23)	1.21 (0.24)	1.20 (0.19)	1.25 (0.25)	0.123	1.40 (0.24)	**< 0.001**

Median (IQR)					*p* ^e^	
PPD 4–5 mm	3.5 (0–10.0)	8.0 (3.0–16.0)	17.5 (8.2–31.5)	13.0 (6.0–20.5)	**< 0.001**	—
PPD ≥ 6 mm	0 (0–0)	0 (0–1.0)	2.5 (1.0–8.0)	2.0 (0–7.0)	**< 0.001**	—
BOP (%)	23.5 (17.0–39.7)	32.0 (21.0–42.5)	36.0 (22.0–51.7)	45.0 (29.5–63.5)	**< 0.001**	—
Number of teeth	26.5 (18.7–28.0)	25.0 (22.0–28.0)	26.0 (25.0–27.0)	18.0 (12.0–21.5)	**< 0.001**	—

*Note:* Edentulous were compared to dentate patients. Statistically significant *p*‐values are bolded.

Abbreviations: BMI, body mass index; BOP, bleeding on probing; CAD, coronary artery disease; DM, diabetes mellitus; GlycA, glycoprotein acetyls; PPD, probing pocket depth.

^a^ *p*‐values calculated using Pearson's Chi‐square test.

^b^ Includes current and former smokers.

^c^ *p*‐values calculated using one‐way ANOVA.

^d^ *p*‐values calculated using independent‐samples *t*‐test (equal variances assumed).

^e^ *p*‐values were calculated using the Kruskal–Wallis test.

As shown in Figure S1, several concentrations of lipid compounds differed between periodontitis stage or dentition state. Cer(d18:1/16:0)/PC(16:0/22:5), Cer(d18:1/18:0)/PC(16:0/22:5), Cer(d18:1/24:1)/Cer(d18:1/24:0) and Cer(d18:1/24:1)/PC(16:0/22:5) increased with increasing stage, whereas PC(14:0/22:6) and PC(16:0/22:5) decreased. Higher concentrations of Cer(d18:1/16:0), Cer(d18:1/16:0)/PC(14:0/22:6), Cer(d18:1/16:0)/PC(16:0/22:5), Cer(d18:1/18:0), Cer(d18:1/18:0)/Cer(d18:1/24:0), Cer(d18:1/18:0)/PC(14:0/22:6), Cer(d18:1/18:0)/PC(16:0/22:5), Cer(d18:1/24:1)/PC(14:0/22:6) and Cer(d18:1/24:1)/PC(16:0/22:5) were observed in edentulous participants compared to dentate ones. Periodontitis grade displayed higher concentrations of Cer(d18:1/16:0), Cer(d18:1/16:0)/PC(14:0/22:6), Cer(d18:1/16:0)/PC(16:0/22:5), Cer(d18:1/18:0)/PC(16:0/22:5), Cer(d18:1/24:1), Cer(d18:1/24:1)/PC(14:0/22:6) and Cer(d18:1/24:1)/PC(16:0/22:5) (Figure S1). In contrast, PC(14:0/22:6) decreased with higher grade. In addition, PC(16:0/22:5) showed a non‐linear pattern, peaking at Grade B. CERT2 showed a significant positive trend across stages (*p* = 0.011) (Figure 1).

**FIGURE 1:**
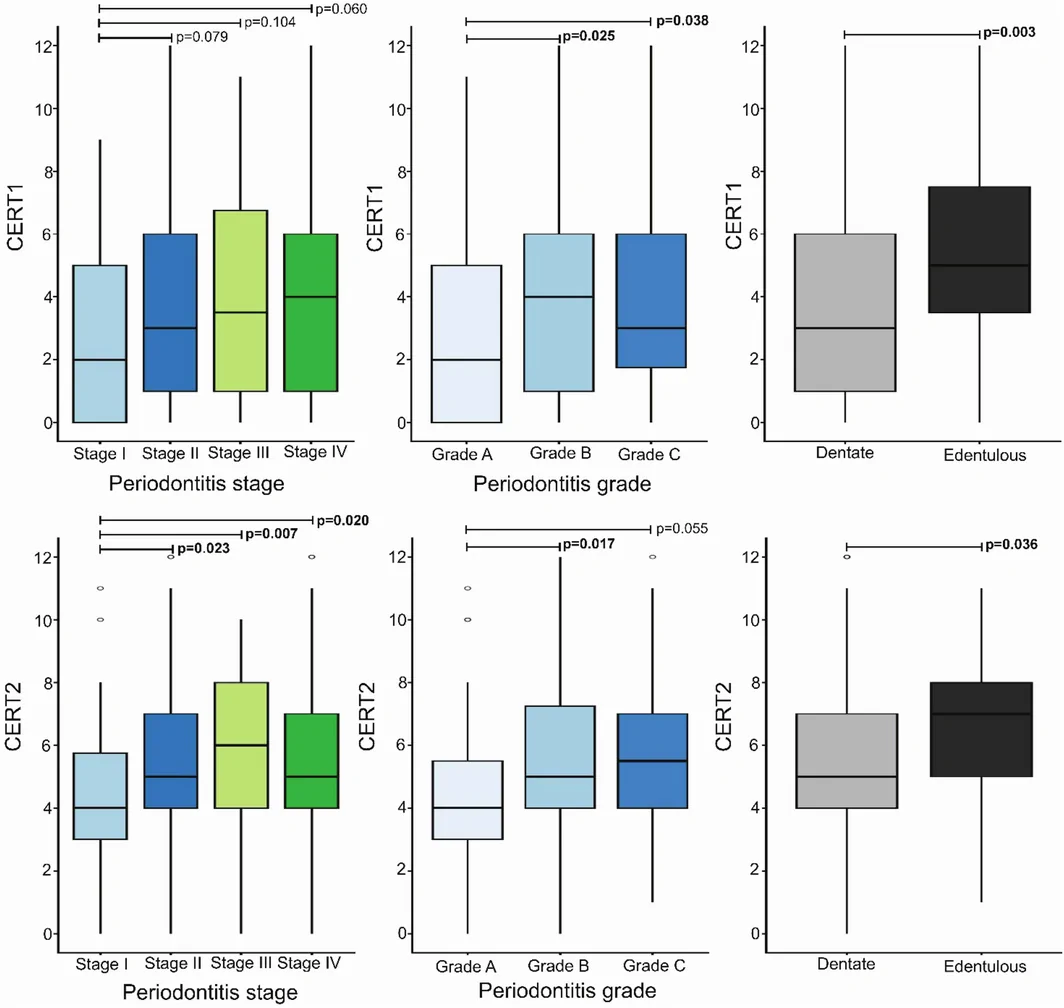
Boxplots showing CERT1 and CERT2 scores (ceramide‐based risk scores) according to periodontitis Stages (I–IV), Grades (A–C) and dentition status (dentate vs. edentulous). Boxes represent the interquartile range (IQR, 25th–75th percentile), with the horizontal line indicating the median. Whiskers show minimum and maximum values. ° indicates outliers (> 1.5 × IQR). *p*‐values above lines indicate pairwise comparisons between groups, calculated using post hoc least significant difference (LSD) test after one‐way ANOVA.

Table 2 presents the results from the multivariate association analyses between lipid compounds and periodontal stages, grades, edentulousness, PPD 4–5 mm and BOP%. After adjusting for age, sex, smoking, BMI and diabetes mellitus, periodontitis stage (Stages 2–4 combined vs. Stage 1) was significantly associated with seven lipid measures. Periodontitis stage was positively associated with Cer(d18:1/16:0)/Cer(d18:1/24:0), Cer(d18:1/16:0)/PC(16:0/22:5), Cer(d18:1/18:0)/Cer(d18:1/24:0), Cer(d18:1/18:0)/PC(16:0/22:5), Cer(d18:1/24:1)/PC(16:0/22:5) and the CERT2 score. In contrast, Stages 2–4 were negatively associated with PC(14:0/22:6) concentrations.

**TABLE 2:** Association of oral parameters with lipid compounds at baseline.

Outcome: lipid species	Stage			Grade			Edentulousness			PPD 4–5 mm			BOP%		
	B	95% CI	FDR	B	95% CI	FDR	B	95% CI	FDR	B	95% CI	FDR	B	95% CI	FDR
Cer(d18:1/16:0)	0.01	−0.2, 0.048	0.57	0.01	−0.03, 0.02	0.15	0.04	0.00, 0.08	**0.044**	0.01	−0.01, 0.03	0.24	0.02	−0.00, 0.02	0.063
Cer(d18:1/16:0)/Cer(d18:1/24:0)	0.02	0.00, 0.04	**0.043**	0.02	0.00, 0.05	**0.048**	0.01	−0.01, 0.03	0.550	0.008	−0.00, 0.00	0.13	0.01	−0.01, 0.01	0.26
Cer(d18:1/16:0)/PC(14:0/22:6)	0.10	−0.01, 9.21	0.07	0.09	0.05	0.12	0.24	0.10, 0.37	**< 0.001**	0.003	−0.06, 0.05	0.93	0.06	0.00, 0.12	**0.043**
Cer(d18:1/16:0)/PC(16:0/22:5)	0.002	0.00, 0.00	**0.039**	0.001	−0.00, 0.01	0.12	0.002	0.00, 0.00	**0.039**	0.00	−0.00, 0.00	0.51	0.00	−0.00, 0.00	0.58
Cer(d18:1/18:0)	0.01	−0.0, 0.03	0.30	0.01	−0.02, 0.01	0.27	0.03	0.01, 0.60	**0.039**	0.002	−0.01, 0.01	0.81	0.01	−0.00, 0.02	0.32
Cer(d18:1/18:0)/Cer(d18:1/24:0)	0.01	0.00, 0.02	**0.010**	0.01	0.00, 0.04	**0.045**	0.01	0.00, 0.02	**0.048**	0.01	0.00, 0.02	**0.040**	0.00	−0.00, 0.01	0.46
Cer(d18:1/18:0)/PC(14:0/22:6)	0.10	−0.01, 0.22	0.07	0.05	−0.13, 0.02	0.28	0.14	0.07, 0.21	**< 0.001**	0.014	−0.01, 0.02	0.43	0.02	−0.01‐0.05	0.18
Cer(d18:1/18:0)/PC(16:0/22:5)	0.001	0.00, 0.00	**0.025**	0.001	0.00, 0.00	**0.025**	0.001	0.00, 0.00	**0.004**	0.00	0.00, 0.00	**0.040**	0.00	−0.00, 0.00	0.45
Cer(d18:1/24:0)	−0.14	−0.38, 0.10	0.25	−0.09	−0.26, 0.08	0.28	0.10	−0.16, 0.36	0.55	0.12	−0.01, 0.24	0.12	0.03	−0.10, 0.16	0.62
Cer(d18:1/24:1)	0.04	−0.13, 0.20	0.67	0.04	−0.15, 0.08	0.12	0.13	−0.04, 0.31	0.21	0.04	−0.05, 0.12	0.51	0.04	−0.05, 0.13	0.36
Cer(d18:1/24:1)/Cer(d18:1/24:0)	0.06	−0.00, 0.13	0.06	0.03	−0.02, 0.07	0.30	0.02	−0.05, 0.09	0.59	−0.030	−0.06, 0.01	0.141	0.01	−0.03, 0.04	0.74
Cer(d18:1/24:1)/PC(14:0/22:6)	0.32	−0.06, 0.71	0.10	0.18	−0.09, 0.90	0.42	0.85	0.39, 1.30	**< 0.001**	0.16	0.02, 0.18	**0.028**	0.20	0.02, 0.41	**0.047**
Cer(d18:1/24:1)/PC(16:0/22:5)	0.005	0.00, 0.01	**0.040**	0.004	0.00, 0.01	**0.042**	0.007	0.00, 0.01	**0.037**	0.00	−0.00, 0.00	0.51	0.00	−0.00, 0.00	0.79
PC(14:0/22:6)	−0.25	−0.47, −0.01	**0.037**	−0.05	−0.15, 0.13	0.18	−0.19	−0.44, 0.04	0.19	−0.14	−0.33, −0.03	**0.026**	−0.10	−0.22, −0.02	**0.036**
PC(16:0/16:0)	−1.02	−2.58, 0.54	0.20	−0.70	−1.36, 0.54	0.39	−0.58	−2.26, 1.04	0.55	−0.11	−0.95, 0.73	0.79	0.06	−0.81, 0.92	0.84
PC(16:0/22:5)	−3.01	−6.73, 0.70	0.11	−2.08	−4.66, 0.51	0.054	−0.55	−4.50, 3.40	0.78	−0.81	−1.22, 2.43	0.55	−1.40	−3.20, −0.67	**0.044**
CERT1	0.85	−0.28, 1.98	0.14	1.20	0.02–2.00	**0.048**	1.46	0.24, 2.67	**0.044**	0.048	−0.26, 0.57	0.81	0.24	−0.39, 0.88	0.45
CERT2	0.92	0.02–1.83	**0.045**	0.93	0.02–1.90	**0.044**	0.74	−0.21, 1.71	0.21	0.13	−0.66, 0.32	0.62	0.15	−0.36, 0.60	0.56

*Note:* Linear regression models were used for baseline analyses. Comparisons were following: periodontal Stages 2–4 versus Stage 1, Grades B and C versus Grade A, complete edentulousness to the presence of teeth, PPD 4–5 mm > 5 versus ≤ 5, BOP% tertiles 2–3 versus 1. All models were adjusted for age, sex (male/female), smoking (no/former/current), BMI and diabetes mellitus (no/yes). Concentrations are in μmol/L. Statistically significant *p*‐values are bolded.

Periodontitis grade (Grades B and C combined vs. Grade A) was also associated with six lipid measures with mainly similar profiles as periodontitis stage. However, periodontitis grade was associated with both CERT1 and CERT2 (Table 2). Edentulousness presented significant associations with 10 lipid compounds (Table 2). Especially strong associations were observed for Cer(d18:1/16:0)/PC(14:0/22:6), Cer(d18:1/16:0)/PC(14:0/22:6), Cer(d18:1/18:0)/PC(16:0/22:5) and Cer(d18:1/24:1)/PC(14:0/22:6). Clinical parameters PPD ≥ 4 mm and BOP were both associated with four lipid measures (Table 2).

Mediation analyses indicated that systemic inflammation, assessed by GlycA, partly mediated the associations between periodontal measures and lipid risk scores (Figure 2, Table S3). For periodontitis stage, significant indirect effects were observed for CERT1 (ACME = 0.403, *p* = 0.046) and CERT2 (ACME = 0.292, *p* = 0.064), although the latter did not reach statistical significance. These corresponded to approximately 46% and 34% of the total effects mediated by GlycA. For periodontitis grade, GlycA significantly mediated the associations with both CERT1 (ACME = 0.386, *p* = 0.020) and CERT2 (ACME = 0.281, *p* = 0.040), corresponding to approximately 35% and 33% of the total effects. Similarly, significant indirect effects were observed for dentition status on CERT1 (ACME = 0.942, *p* < 0.001) and CERT2 (ACME = 0.663, *p* < 0.001), equivalent to 59% and 95% of the total effects. Direct effects were generally weaker and mostly non‐significant, except for the association between periodontitis stage and CERT2 (ADE = 0.829, *p* = 0.015) and between periodontitis grade and CERT2 (ADE = 0.758, *p* = 0.047) (Figure 3, Table S4). Additionally, GlycA significantly mediated the associations between periodontal measures and several individual ceramide and phospholipid species, although the indirect effects were generally modest in magnitude (Figure 2, Table S3).

**FIGURE 2:**
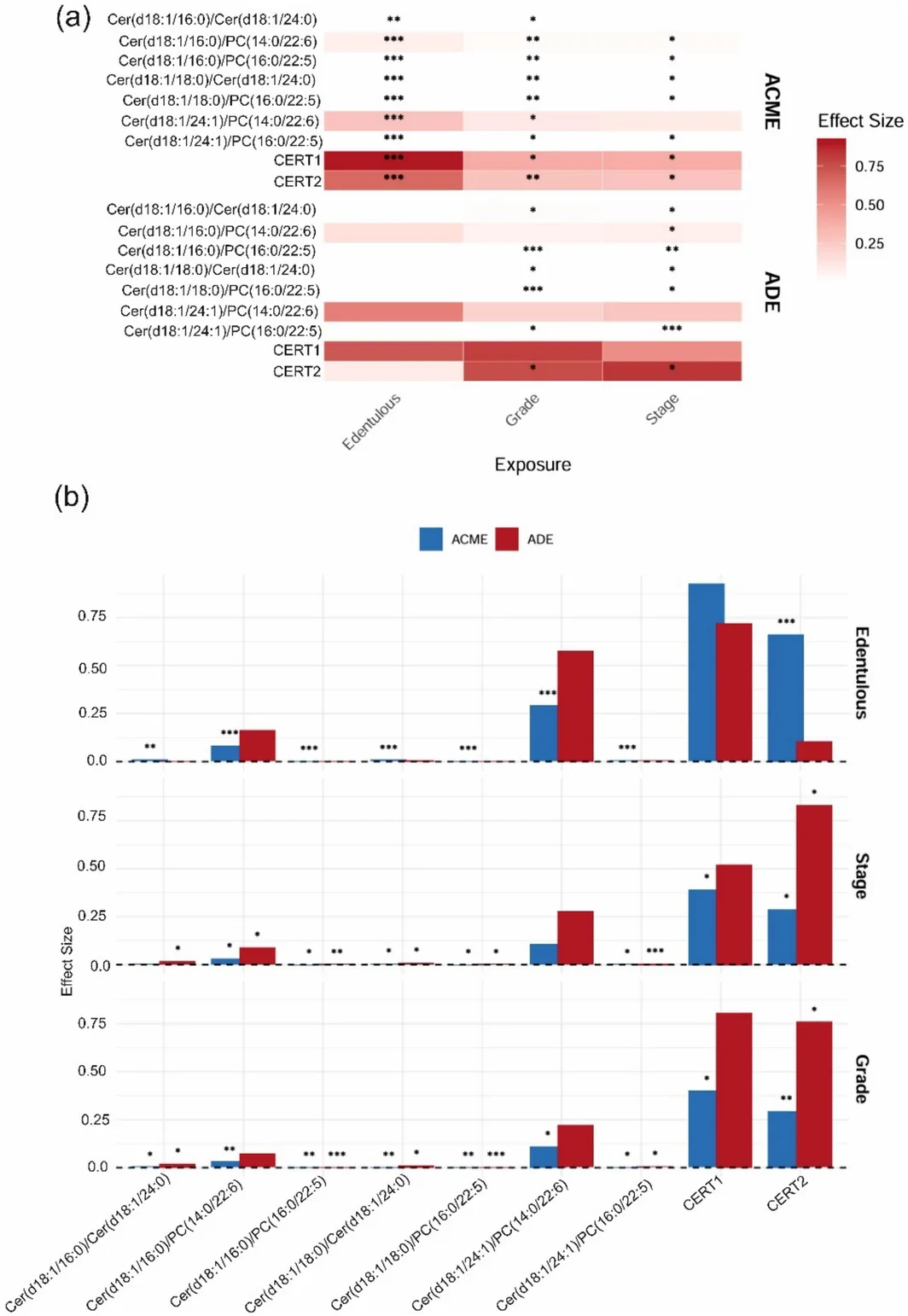
Mediation analysis of oral health measures and ceramide‐related lipid outcomes through GlycA. (a) Heatmap of the average causal mediation effects (ACME) and average direct effects (ADE) for periodontitis stage, periodontitis grade and edentulism across ceramide‐related lipid outcomes. (b) Bar plots showing the estimated indirect (ACME, blue) and direct (ADE, red) effects of each oral health measure on the lipid outcomes. Significance indicated by asterisks (*p < 0.05, **p < 0.01, *p < 0.001).

**FIGURE 3:**
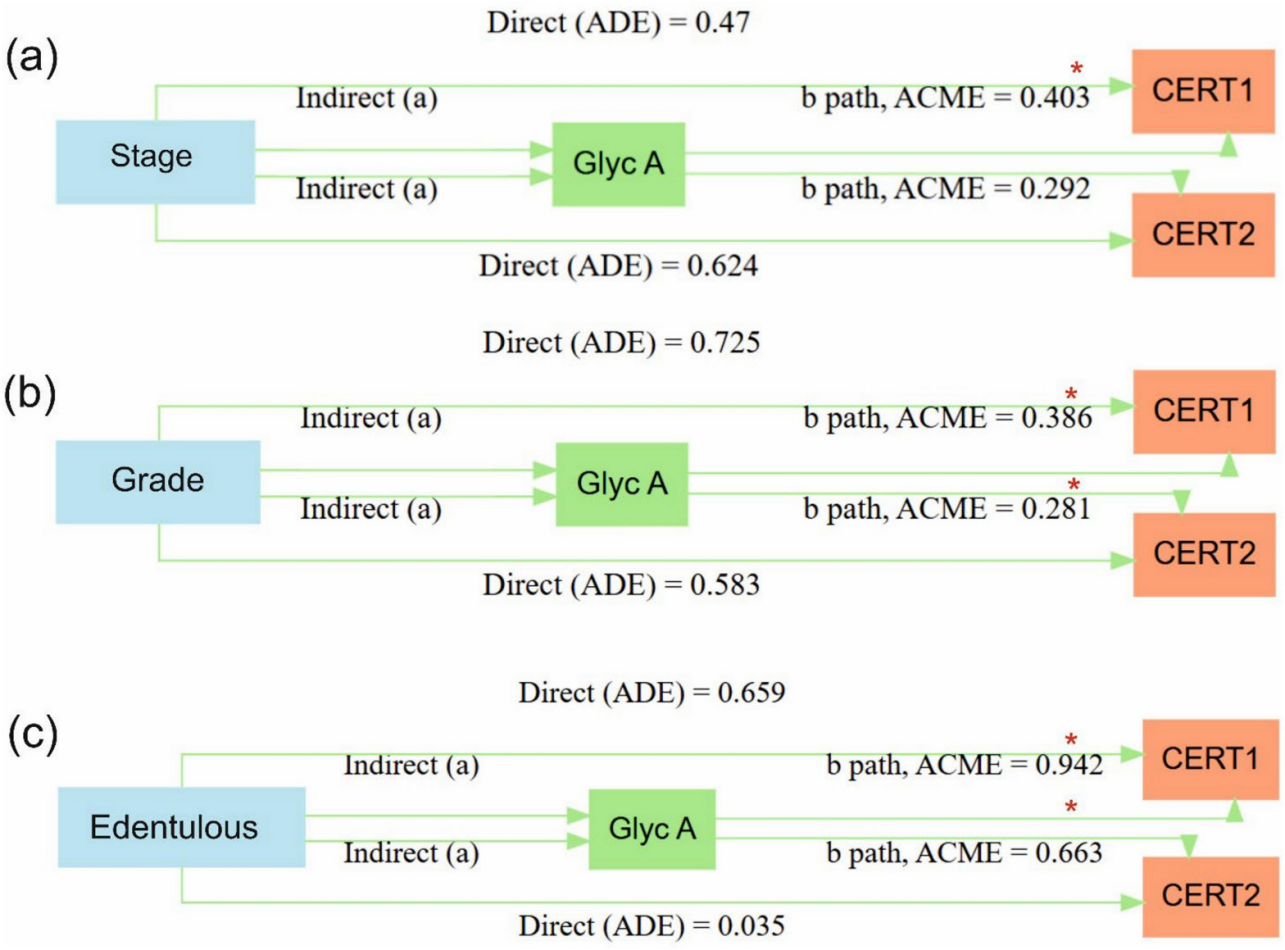
Causal mediation pathways of GlycA linking oral health exposures with lipid‐based cardiovascular risk scores (CERT1, CERT2). Arrows indicate mediation through Glyc A (ACME) and direct effects (ADE). Significance is indicated by asterisks (*p < 0.05).

In the follow‐up dataset (Table 3), frequency of smoking was higher among those with severe periodontal progression, while other characteristics did not differ between the groups. Participants who lost more than one tooth due to periodontitis showed significantly lower levels of Cer(d18:1/24:1) and PC(16:0/16:0) compared with those who lost 0–1 tooth (Figure S2). Participants with rapidly progressing periodontitis exhibited significantly lower concentrations of Cer(d18:1/24:0) and PC(14:9/22:6) than those with slow progression. The same lipid measures were associated with periodontitis progression in multivariate analyses (Table 4).

**TABLE 3:** Baseline demographic and clinical characteristics of participants according to periodontitis progression in the follow‐up.

	0–1 lost tooth *n* = 49	> 1 lost tooth *n* = 64	*p* ^a^	No or slow progression *n* = 81	Severe progression *n* = 32	*p* ^a^
	*N* (%)			*N* (%)		
Females	19 (35.2)	27 (38.6)	0.699	38 (41.8)	8 (24.2)	0.074
Smokers^b^	23 (42.6)	37 (52.9)	0.257	39 (42.9)	21 (63.6)	**0.041**
CAD	30 (56.6)	48 (68.6)	0.172	55 (61.1)	23 (69.7)	0.381
Hypertension	33 (62.3)	44 (62.9)	0.946	54 (60.0)	23 (69.7)	0.325
DM I or II	7 (13.2)	15 (21.4)	0.239	14 (15.6)	8 (24.2)	0.265
Dyslipidemia	41 (77.4)	58 (84.1)	0.348	73 (81.1)	26 (81.3)	0.986

Mean (SD)			*p* ^c^	Mean (SD)		*p* ^c^
Age (years)	59.1 (8.18)	61.4 (7.86)	0.111	60.1 (7.73)	61.1 (8.98)	0.577
BMI (kg/$m^{2}$)	27.7 (4.59)	28.1 (4.89)	0.668	27.6 (4.11)	28.9 (6.13)	0.183
GlycA (μmol/L)	1.17 (0.22)	1.16 (0.19)	0.737	1.16 (0.20)	1.20 (0.22)	0.386

Median (IQR)			*p* ^d^	Median (IQR)		*p* ^d^
PPD 4–5 mm	6.5 (4.0–18.0)	12.5 (6.7–20.2)	**0.034**	10.0 (4.0–17.0)	16.0 (9.5–16.0)	**0.007**
PPD ≥ 6 mm	0 (0–2.0)	1 (0–7.0)	**0.006**	0 (0–2.0)	5 (1.0–15.0)	**< 0.001**
BOP (%)	29.5 (22.0–43.0)	42.0 (27.0–56.5)	**0.003**	30.0 (22.0–43.0)	49.0 (34.0–63.5)	**< 0.001**
Number of teeth	26.0 (22.7–28.0)	24.0 (20.0–27.0)	**0.037**	26.0 (22.0–28.0)	23.0 (17.5–23.0)	**0.007**

*Note:* Severe progression of periodontitis during the follow‐up was defined as radiographical progression of bone loss in the worst site > 4 mm during follow‐up or at least one tooth lost due to periodontitis versus slowly progressing periodontitis. Tooth loss was defined as those with 0–1 versus > 1 teeth lost.

Abbreviations: BMI, body mass index; BOP, bleeding on probing; CAD, coronary artery disease; DM, diabetes mellitus; GlycA, glycoprotein acetyls; IQR, interquartile range; PPD, probing pocket depth. Statistically significant *p*‐values are bolded.

^a^ *p*‐values calculated using Pearson's Chi‐square.

^b^ Includes current and former smokers.

^c^ *p*‐values calculated using independent‐samples *t*‐test (equal variances assumed).

^d^ *p*‐values calculated using the Mann–Whitney test.

**TABLE 4:** Associations of baseline lipid compounds with progression of periodontitis in the follow‐up.

Exposure: lipid species	Follow‐up outcome					
	Severe progression			Loosing teeth		
	RR	95% CI	FDR	RR	95% CI	FDR
Cer(d18:1/16:0), (μmol/L)	0.24	0.00–1.69	0.78	0.03	0.00–1.07	0.80
Cer(d18:1/16:0)/Cer(d18:1/24:0)	1.73	0.26–1.75	0.45	0.99	0.01–1.06	0.55
Cer(d18:1/16:0)/PC(14:0/22:6)	1.20	0.52–1.62	0.75	1.27	0.41–1.01	0.58
Cer(d18:1/16:0)/PC(16:0/22:5)	1.75	0.00–1.76	0.68	0.11	0.00–0.92	0.96
Cer(d18:1/18:0), (μmol/L)	0.53	0.00–1.77	0.78	0.07	0.00–1.07	0.57
Cer(d18:1/18:0)/Cer(d18:1/24:0)	1.75	0.04–1.75	0.25	1.34	0.01–1.36	0.53
Cer(d18:1/16:0)/PC(14:0/22:6)	1.23	0.17–1.73	0.72	1.25	0.11–1.07	0.68
Cer(d18:1/18:0)/PC(16:0/22:5)	1.06	0.00–1.60	0.55	1.04	0.00–1.08	0.80
Cer(d18:1/24:0), (μmol/L)	0.63	0.35–0.95	**0.027**	0.61	0.34–1.00	0.054
Cer(d18:1/24:1), (μmol/L)	0.82	0.37–1.30	0.78	0.44	0.15–0.92	**0.045**
Cer(d18:1/24:1)/Cer(d18:1/24:0)	1.61	0.87–1.75	0.17	0.82	0.13–0.97	0.81
Cer(d18:1/24:1)/PC(14:0/22:6)	1.05	0.86–1.23	0.60	1.01	0.74–1.17	0.83
Cer(d18:1/24:1)/PC(16:0/22:5)	1.65	0.00–1.67	0.78	0.75	0.00–1.12	0.68
PC(14:0/22:6), (μmol/L)	0.71	0.41–1.05	0.17	0.74	0.45–1.06	0.16
PC(16:0/16:0), (μmol/L)	0.96	0.91–1.01	0.11	0.92	0.86–0.98	**0.046**
PC(16:0/22:5), (μmol/L)	0.99	0.97–1.01	0.25	0.98	0.96–1.01	0.43
CERT1	1.01	0.95–1.08	0.66	0.97	0.88–1.05	0.51
CERT2	1.01	0.93–1.10	0.78	0.97	0.88–1.08	0.71

*Note:* Logistic regression models for periodontitis progression. Severe progression of periodontitis during the follow‐up was defined as progression of RBL in the worst site > 4 mm during follow‐up or at least one tooth lost due to periodontitis vs. slowly progressing periodontitis. Tooth loss was defined as those with 0–1 versus > 1 teeth lost. All models were adjusted for age, sex (male/female), smoking (no/former/current), BMI and diabetes mellitus (no/yes). Relative risks (RRs) were estimated from reported odds ratios (ORs) using the risk, which was defined as the proportion of individuals experiencing the outcome. Statistically significant *p*‐values are bolded.

## 4 Discussion

In this study, systemic lipidomic profiling revealed consistent alterations in circulating ceramides and ceramide:PC ratios, with multiple ratios showing significant positive associations with increasing periodontitis stage and grade. Individuals without teeth showed elevated concentrations across several lipid species, supporting a link between these lipid alterations and a history or burden of periodontitis. Overall, these findings highlight a clear relationship between disrupted systemic lipid profiles and periodontal disease severity.

Individual ceramide species containing palmitic acid (16:0), stearic acid (18:0), oleic acid (24:1) or lignoceric acid (24:0) did not associate significantly with periodontitis stage or grade when covariates were considered, but Cer(d18:1/16:0) and Cer(d18:1/18:0) were higher in edentulous subjects. In contrast, all measured phosphatidylcholine species displayed an inverse association with both periodontitis stage and grade, with the strongest associations observed for PCs containing 22‐carbon polyunsaturated fatty acids, specifically PC(14:0/22:6) and PC(16:0/22:5), suggesting a potential protective or anti‐inflammatory lipid profile. These lipid species displayed negative estimates also with rapid progression of periodontitis or losing teeth in the follow‐up, but the differences mainly attenuated to non‐significant when considering confounders. The phosphatidylcholines containing long‐chain PUFAs are likely derived from diet, especially fish oils. Docosahexaenoic acid (DHA, 22:6) and docosapentaenoic acid (DPA, 22:5) are precursors of lipid mediators that actively resolve inflammation (Basil and Levy 2016). In a large recent study using metabolomics, relative proportions of omega‐3 fatty acids were found to be inversely associated with BOP and alveolar bone loss, but also with apical rarefactions and caries (Salminen et al. 2024). A systematic review suggested that supplementation with omega‐3 PUFA might increase the effectiveness of a non‐surgical periodontal treatment (Miroult et al. 2023). These observations are consistent with our findings, where PC(14:0/22:6) and PC(16:0/22:5) were inversely associated with periodontitis severity. However, our study does not allow conclusions about causal mechanisms or direct effects on inflammatory resolution within periodontal tissues.

The ratio of Cer(d18:1/16:0) to Cer(d18:1/24:0) and PC(16:0/22:5), the ratio of Cer(d18:1/18:0) to Cer(d18:1/24:0) and PC(16:0/22:5), and the ratio of Cer(d18:1/24:1) to PC(16:0/22:5), were directly associated with both periodontitis stage and grade. The biological behaviour of ceramides is strongly dependent on the length of the fatty acid chain: Cer(d18:1/16:0) can be considered pro‐inflammatory, promoting oxidative stress and inducing cytokines, whereas Cer(d18:1/24:0) is anti‐inflammatory, maintaining barrier integrity and limiting stress responses (Hannun and Obeid 2018). The ratio with PC(16:0/22:5) may act as a metabolic index reflecting the inflammatory versus resolving lipid balance (Gaggini et al. 2022). Bacterial lipopolysaccharide (LPS)—an important inflammatory agent in periodontitis (Pussinen et al. 2022)—drives especially short‐chain ceramide accumulation, whereas ceramides amplify LPS responses via TLR4 and ceramide microdomains (Gaggini et al. 2022), forming a vicious circle fuelling chronic inflammation. Thus, in periodontitis, the high ceramide/PC ratios may designate inflammation, oxidative stress and tissue damage. However, the ratios were not associated with severe progression of periodontitis or tooth loss in the follow‐up. The lack of longitudinal associations suggests that these lipid species may not be stable long‐term predictors of disease progression. One plausible explanation is that circulating lipid profiles, particularly ceramides and phospholipids, are dynamic and may reflect short‐term metabolic and inflammatory states rather than cumulative exposure over extended periods such as 12 years.

Edentulism and tooth loss reflect cumulative oral health history; however, they are not specific indicators of periodontitis severity, as they may also result from caries, trauma, prosthodontic decisions or mixed aetiologies. Nevertheless, both have been associated with systemic conditions, including cardiovascular diseases, diabetes, increased mortality (Liljestrand et al. 2015) and dementia (Fang et al. 2018). In a large metabolomics study, the number of teeth was significantly associated with 60 out of 157 (38%) measured metabolites related to systemic inflammation and cardiometabolic risk, and baseline tooth loss predicted longitudinal changes in lipoprotein‐ and fatty acid‐related measures (Halme et al. 2025). In the present study, edentulism was positively associated with several lipid species, particularly Cer(d18:1/16:0)/PC(14:0/22:6) and Cer(d18:1/24:1)/PC(14:0/22:6), suggesting a shift towards a more pro‐inflammatory lipid profile characterised by increased ceramide burden and reduced ω‐3‐containing phosphatidylcholines. This pattern may reflect long‐term systemic alterations linked to chronic inflammatory exposure, although the specific contribution of periodontitis cannot be isolated in edentulous individuals. Consistent with our results, tooth loss has been associated with systemic inflammation, as indicated by increasing C‐reactive protein (CRP) concentrations with advancing tooth loss (Liljestrand et al. 2015).

Previously, the prognostic test score, named the coronary event risk test (CERT) combining different ceramides, has been shown to be incomparable in predicting cardiovascular risk compared to traditional risk markers (Hilvo et al. 2020). Ceramides are bioactive lipids that form signalling platforms in lipid rafts and regulate cellular functions through phosphatases and downstream targets (Canals and Hannun 2024). Acting as hubs in lipid metabolism, facilitating endothelial lipid uptake, mediating lipotoxicity and promoting arterial dysfunction, ceramides have emerged as strong predictors of cardiovascular event risk (Hilvo et al. 2020). In the present study, CERT1 and CERT2 were associated with periodontitis stage, grade or edentulousness, but they were not significantly associated with severe periodontitis progression or tooth loss over 12 years. Although ceramides influence multiple inflammatory processes, their impact on periodontitis progression was not evident and they might not causally contribute to disease worsening. Nevertheless, this study did not directly assess inflammatory resolution pathways in periodontal tissues; thus no conclusions can be drawn regarding the junderlying mechanistic processes at the tissue level. However, periodontitis patients with elevated levels of these bioactive lipids in circulation may experience enhanced endothelial dysfunction and atherogenesis.

Our findings indicate that the relationship between periodontitis and lipid metabolism varies according to the periodontal construct examined and involves both direct and inflammation‐mediated pathways. Periodontitis stage showed significant GlycA‐mediated associations with CERT1 and several ceramide‐related lipid ratios, whereas the indirect effect on CERT2 was of similar magnitude but did not reach statistical significance. Direct effects remained significant for some lipid outcomes and for CERT2, but not for CERT1, suggesting that systemic inflammation contributes importantly to these associations. Similarly, periodontitis grade showed consistent evidence of partial mediation, with significant indirect effects of GlycA on both CERT1 and CERT2 as well as several ceramide ratios, supporting its role as a marker of systemic susceptibility and inflammatory burden (Chiesa et al. 2022; Salminen et al. 2024). In contrast, edentulism exhibited a markedly different pattern, with GlycA acting as the dominant mediator of the association with lipid measures. Indirect effects were particularly pronounced for CERT1 and CERT2, while direct effects were weak and non‐significant, suggesting that the relationship between tooth loss and adverse lipid profiles is largely driven by systemic inflammation. This finding underscores the pronounced inflammatory component associated with long‐standing tooth loss and its systemic metabolic consequences reported in earlier studies (Liljestrand et al. 2015; Halme et al. 2025).

The main limitation of the study is the restricted sample size, including 473 patients in the cross‐sectional analyses and 117 participating in the follow‐up. The relatively high attrition rate at follow‐up may introduce selection bias and reduce statistical power; therefore, longitudinal findings should be interpreted with caution. In addition, stratifying the cohort into subgroups further reduced statistical power, increasing the likelihood of imprecise or unstable estimates. Multiple comparisons of different lipid species increased the potential for type I error. Several analyses displayed significant but weak associations, with FDR (false discovery rate) values ranging between 0.049 and 0.01, which should be interpreted with caution. Using Bonferroni correction, a more stringent amendment for multiple testing, only *p*‐values ≤ 0.003 would have met the threshold for statistical significance. Another important limitation is that lipidomic profiles were measured at baseline in a cardiology cohort undergoing coronary angiography, whereas periodontal outcomes were assessed at baseline and approximately 12 years later without accounting for intervening cardiovascular treatments. As circulating lipid levels are highly responsive to medical therapy, subsequent treatments may have influenced or attenuated the observed longitudinal associations. In addition, information on periodontal care during follow‐up was unavailable; oral treatments such as periodontal therapy, maintenance care or extractions may have modified both periodontal inflammation and disease progression. Additionally, the age and gender distribution of the participants reflected that of individuals in Finland who typically present with cardiological symptoms, a group that also has poorer oral health than the general population (Buhlin et al. 2011). Intraoral radiographs and information on the reasons for tooth extractions were unfortunately not available; therefore, the definition of periodontitis progression was only based on findings from panoramic radiographs. Nonetheless, the strengths of our study—particularly its novelty and the availability of longitudinal follow‐up data—should be acknowledged.

## 5 Conclusion

In summary, periodontitis stage and grade, as well as edentulousness, were associated with several circulating bioactive lipid species, as reflected by altered ceramide and phospholipid profiles. Our findings indicate that systemic inflammation mediates the relationship between periodontitis and circulating lipid species, linking periodontitis to broader disturbances in lipidomic homeostasis. As the first study to apply lipidomics in periodontal research, these results expand our understanding of systemic lipid alterations associated with periodontitis and their potential relevance to the increased cardiovascular risk observed in affected individuals.

## Funding

The work received funding (through P.P.) from the Research Council of Finland (340750 and 369310), the Sigrid Juselius foundation, the Finnish Dental Society Apollonia, the Paulo foundation and Novo Nordisk (NNF230C0084173).

## Ethics Statement

The research plans were approved by the Ethical committee of Helsinki University Hospital per permissions HUS 426/E5/05 and HUS 152/2016. All participants signed an informed consent both in the baseline and follow‐up examinations.

## Conflicts of Interest

The authors declare no conflicts of interest.

## Supporting information


**Table S1:** Baseline demographic and clinical characteristics of participants according to their participation in the follow‐up.
**Table S2:** Demographic and clinical characteristics of participants according to periodontitis grade A–C.
**Table S3:** Mediation of the effect of grade, stage and edentulism on GlycA across two adjustment models.
**Table S4:** Estimated ACME (indirect), ADE (direct) and total effects of periodontitis stage, periodontitis grade and dentition status on lipid outcomes (CERT1 and CERT2) mediated by GlycA. Effect sizes with *p*‐values are shown.
**Figure S1:** Serum ceramide (Cer) and phosphatidylcholine (PC) concentrations and their ratios according to (a) periodontitis stage (I–IV), (b) grade (A–C) and (c) dentition status.
**Figure S2:** Serum ceramide (Cer) and phosphatidylcholine (PC) concentrations and their ratios according to (a) tooth loss category and (b) periodontitis progression rate.

## Data Availability

The data that support the findings of this study are available on request from the corresponding author. The data are not publicly available due to privacy or ethical restrictions.
