# Supplementary material for: Systemic Lipidomics Identifies Altered Ceramide–Phospholipid Balance Associated With Periodontitis Severity: A Cross‐Sectional Study With 12‐Year Follow‐Up
Source: J Clin Periodontol. 2026 Aug 2;53(12):1773–84. doi: 10.1111/jcpe.70182 (PMC13751313; doi:10.1111/jcpe.70182)
Supplement: Supplementary file 1 [file JCPE-53-1773-s001.docx]

**Table S1.** Baseline demographic and clinical characteristics of participants according to their participation in the follow-up.

|  |  | **Participated** | **Did not participate** |  |
| --- | --- | --- | --- | --- |
|  |  | **N (%)** | | **p-value†** |
| Females |  | 52 (38.5) | 126 (33.8) | 0.323 |
| Smoking | No | 71 (52.6) | 168 (45.4) | 0.325 |
|  | Ex | 49 (36.3) | 159 (42.7) |  |
|  | Current | 15 (11.1) | 45 (12.1) |  |
| CAD |  | 86 (64.2) | 267 (72.0) | 0.092 |
| Hypertension |  | 85 (63.4) | 237 (64.1) | 0.898 |
| DM I or II |  | 25 (18.7) | 93 (25.3) | 0.119 |
| Dyslipidemia |  | 107 (80.5) | 297 (80.3) | 0.964 |
| Periodontitis |  |  |  |  |
|  | Stage I | 11 (8.2) | 31 (8.4) | 0.167 |
|  | Stage II | 71 (53.0) | 154 (41.6) |  |
|  | Stage III | 16 (11.9) | 44 (11.9) |  |
|  | Stage IV | 30 (22.4) | 115 (31.1) |  |
|  | Edentulous | 6 (4.5) | 26 (7.0) |  |
|  | Grade A | 11 (8.5) | 35 (10.2) | 0.627 |
|  | Grade B | 103 (79.8) | 278 (80.8) |  |
|  | Grade C | 15 (11.6) | 31 (9.0) |  |
|  |  | **Mean (SD)** | | **p-value¶** |
| Age (years) |  | 60.6 (8.4) | 64.3 (9.2) | **<0.001** |
| BMI (kg/$m^{2}$) |  | 28.0 (4.9) | 27.8 (5.1) | 0.584 |
| GlycA (µmol/l) |  | 1.18 (0.21) | 1.24 (0.24) | **0.013** |
|  |  | **Median (IQR)** | | **p-value**‖ |
| PPD 4-5 mm |  | 11 (5.0-19.5) | 9.0 (3.0-19.0) | 0.162 |
| PPD ≥ 6 mm |  | 0 (0-4.0) | 0 (0-3.0) | 0.248 |
| BOP (%) |  | 34 (24.0-47.5) | 34 (21.5-51.0) | 0.508 |
| Number of teeth |  | 25 (22.0-28.0) | 23 (16.0-27.0) | **<0.001** |

† P-values calculated using Pearson’s Chi-square.

¶ P-values calculated using independent-samples t-test (equal variances assumed).

‖ P-values calculated using Mann-Whitney test.

CAD, coronary artery disease; DM, diabetes mellitus; BMI, body mass index; GlycA, glycoprotein acetyls

**Table S2.** Demographic and clinical characteristics of participants according to periodontitis grade (A-C)

|  | **Grade A**  **n=42** | **Grade B**  **n=353** | **Grade C**  **n=44** | **p-value †** |
| --- | --- | --- | --- | --- |
| **N (%)** | | | |  |
| Females | 19 (45.2) | 124 (35.2) | 9 (20.5) | **0.049** |
| Smokers ‡ | 12 (28.6) | 191 (54.3) | 27 (61.4) | **0.003** |
| CAD | 23 (54.8) | 237 (67.5) | 37 (84.1) | **0.013** |
| Hypertension | 19 (45.2) | 233 (66.6) | 26 (59.1) | **0.020** |
| DM I or II | 6 (15.0) | 82 (23.4) | 12 (27.9) | 0.357 |
| Dyslipidemia | 30 (71.4) | 280 (80.0) | 38 (88.4) | 0.149 |
| **Mean (SD)** | | | | **p-value §** |
| Age (years) | 58.4 (11.3) | 63.6 (8.8) | 62.6 (7.9) | **0.002** |
| BMI (kg/$m^{2}$) | 27.6 (4.8) | 28.0 (5.2) | 27.0 (4.6) | 0.430 |
| GlycA (µmol/l) | 1.14 (0.18) | 1.22 (0.23) | 1.30 (0.28) | **0.012** |
| **Median (IQR)** | | | | **p-value^¶^** |
| PPD 4-5 mm | 3.0 (1.0-8.5) | 10.0 (4.0-19.0) | 18.5 (12.0-34.2) | **< 0.001** |
| PPD ≥ 6 mm | 0 (0-0) | 0 (0-2.0) | 7.0 (3.0-20.2) | **< 0.001** |
| BOP (%) | 26.0 (17.0-39.7) | 33.0 (22.0-48.0) | 55.5 (38.2-71.0) | **< 0.001** |
| Number of teeth | 26.0 (17.0-39.7) | 24.0 (22.0-48.0) | 19.0 (38.2-71.0) | **< 0.001** |

† P-values calculated using Pearson’s Chi-square.

‡ Includes current and former smokers.

§ P-values calculated using One-way ANOVA.

¶ P-values calculated using Kruskal-Wallis test.

CAD, coronary artery disease; DM, diabetes mellitus; BMI, body mass index; GlycA, glycoprotein acetyls

**Table S3.** Mediation of the effect of grade, stage and edentulism on GlycA across two adjustment models

| **Exposure** | **Outcome** | **ACME (95% CI)** | **ADE (95% CI)** | **Total effect (95% CI)** |
| --- | --- | --- | --- | --- |
| **Model 1: adjusted for age and sex** | | | |  |
| Dentition status | Cer(d18:1/16:0)/Cer(d18:1/24:0) | 0.008 [0.001, 0.016]* | 0.001 [-0.020, 0.025] | 0.009 [-0.011, 0.034] |
|  | Cer(d18:1/16:0)/PC(14:0/22:6) | 0.086 [0.040, 0.144]* | 0.171 [-0.058, 0.423] | 0.257 [-0.010, 0.527] |
|  | Cer(d18:1/16:0)/PC(16:0/22:5) | 0.001 [0.000, 0.002]* | 0.001 [-0.001, 0.003] | 0.002 [-0.000, 0.005] |
|  | Cer(d18:1/18:0)/Cer(d18:1/24:0) | 0.009 [0.004, 0.015]* | 0.005 [-0.009, 0.021] | 0.014 [0.000, 0.030] |
|  | Cer(d18:1/18:0)/PC(16:0/22:5) | 0.001 [0.000, 0.001]* | 0.001 [-0.000, 0.002] | 0.001 [0.000, 0.003] |
|  | Cer(d18:1/24:1)/PC(14:0/22:6) | 0.329 [0.150, 0.530]* | 0.604 [-0.204, 1.491] | 0.932 [0.065, 1.962] |
|  | Cer(d18:1/24:1)/PC(16:0/22:5) | 0.004 [0.002, 0.007]* | 0.003 [-0.005, 0.012] | 0.007 [-0.002, 0.017] |
|  | CERT1 | 1.019 [0.526, 1.569]* | 0.661 [-0.669, 1.862] | 1.680 [0.282, 2.973] |
|  | CERT2 | 0.727 [0.368, 1.124]* | 0.070 [-0.799, 0.968] | 0.797 [-0.108, 1.753] |
| Periodontitis grade | Cer(d18:1/16:0)/Cer(d18:1/24:0) | 0.004 [0.000, 0.009]* | 0.012 [-0.003, 0.027] | 0.016 [-0.000, 0.031] |
|  | Cer(d18:1/16:0)/PC(14:0/22:6) | 0.037 [0.010, 0.067]* | 0.055 [-0.021, 0.127] | 0.092 [0.007, 0.166] |
|  | Cer(d18:1/16:0)/PC(16:0/22:5) | 0.000 [0.000, 0.001]* | 0.001 [0.000, 0.002]* | 0.002 [0.001, 0.003] |
|  | Cer(d18:1/18:0)/Cer(d18:1/24:0) | 0.004 [0.001, 0.008]* | 0.008 [0.000, 0.015]* | 0.012 [0.004, 0.021] |
|  | Cer(d18:1/18:0)/PC(16:0/22:5) | 0.000 [0.000, 0.001]* | 0.001 [0.000, 0.001]* | 0.001 [0.001, 0.002] |
|  | Cer(d18:1/24:1)/PC(14:0/22:6) | 0.144 [0.046, 0.253]* | 0.166 [-0.096, 0.433] | 0.310 [0.014, 0.605] |
|  | Cer(d18:1/24:1)/PC(16:0/22:5) | 0.002 [0.001, 0.003]* | 0.004 [0.001, 0.007]* | 0.006 [0.003, 0.009] |
|  | CERT1 | 0.497 [0.190, 0.826]* | 0.653 [-0.171, 1.463] | 1.150 [0.251, 2.062] |
|  | CERT2 | 0.367 [0.122, 0.617]* | 0.581 [-0.099, 1.302] | 0.948 [0.207, 1.711] |
| Periodontitis stage | Cer(d18:1/16:0)/Cer(d18:1/24:0) | 0.004 [0.000, 0.009]* | 0.010 [-0.008, 0.026] | 0.014 [-0.004, 0.030] |
|  | Cer(d18:1/16:0)/PC(14:0/22:6) | 0.038 [0.005, 0.073]* | 0.067 [-0.006, 0.144] | 0.105 [0.024, 0.193] |
|  | Cer(d18:1/16:0)/PC(16:0/22:5) | 0.000 [0.000, 0.001]* | 0.001 [0.000, 0.002]* | 0.002 [0.001, 0.002] |
|  | Cer(d18:1/18:0)/Cer(d18:1/24:0) | 0.004 [0.001, 0.008]* | 0.008 [-0.001, 0.016] | 0.012 [0.002, 0.021] |
|  | Cer(d18:1/18:0)/PC(16:0/22:5) | 0.000 [0.000, 0.001]* | 0.001 [0.000, 0.001]* | 0.001 [0.000, 0.002] |
|  | Cer(d18:1/24:1)/PC(14:0/22:6) | 0.145 [0.023, 0.292]* | 0.209 [-0.095, 0.503] | 0.355 [0.010, 0.686] |
|  | Cer(d18:1/24:1)/PC(16:0/22:5) | 0.002 [0.000, 0.004]* | 0.004 [0.001, 0.008]* | 0.006 [0.003, 0.009] |
|  | CERT1 | 0.508 [0.090, 0.931]* | 0.418 [-0.608, 1.307] | 0.927 [-0.166, 1.922] |
|  | CERT2 | 0.372 [0.082, 0.664]* | 0.633 [-0.166, 1.459] | 1.006 [0.214, 1.917] |
| **Model 2: adjusted for age, sex, smoking status (never, former, current), BMI, and diabetes.** | | | | |
| **Exposure** | **Outcome** | **ACME (95% CI)** | **ADE (95% CI)** | **Total effect (95% CI)** |
| Dentition status | Cer(d18:1/16:0)/Cer(d18:1/24:0) | 0.009 [0.002, 0.018]* | 0.000 [-0.021, 0.025] | 0.009 [-0.013, 0.035] |
|  | Cer(d18:1/16:0)/PC(14:0/22:6) | 0.080 [0.037, 0.138]* | 0.162 [-0.065, 0.407] | 0.242 [0.002, 0.524] |
|  | Cer(d18:1/16:0)/PC(16:0/22:5) | 0.001 [0.000, 0.002]* | 0.001 [-0.001, 0.004] | 0.002 [-0.000, 0.005] |
|  | Cer(d18:1/18:0)/Cer(d18:1/24:0) | 0.007 [0.003, 0.012]* | 0.006 [-0.008, 0.020] | 0.013 [-0.001, 0.028] |
|  | Cer(d18:1/18:0)/PC(16:0/22:5) | 0.001 [0.000, 0.001]* | 0.001 [-0.001, 0.002] | 0.001 [0.000, 0.003] |
|  | Cer(d18:1/24:1)/PC(14:0/22:6) | 0.290 [0.131, 0.499]* | 0.575 [-0.164, 1.327] | 0.865 [0.059, 1.740] |
|  | Cer(d18:1/24:1)/PC(16:0/22:5) | 0.004 [0.002, 0.006]* | 0.003 [-0.004, 0.012] | 0.007 [-0.001, 0.017] |
|  | CERT1 | 0.924 [0.474, 1.454]* | 0.718 [-0.560, 1.885] | 1.642 [0.324, 2.902] |
|  | CERT2 | 0.661 [0.318, 1.044]* | 0.101 [-0.809, 1.032] | 0.762 [-0.207, 1.702] |
| Periodontitis grade | Cer(d18:1/16:0)/Cer(d18:1/24:0) | 0.004 [0.000, 0.009]* | 0.018 [0.006, 0.032]* | 0.022 [0.009, 0.036] |
|  | Cer(d18:1/16:0)/PC(14:0/22:6) | 0.031 [0.007, 0.061]* | 0.072 [-0.002, 0.136] | 0.102 [0.026, 0.177] |
|  | Cer(d18:1/16:0)/PC(16:0/22:5) | 0.000 [0.000, 0.001]* | 0.001 [0.001, 0.002]* | 0.002 [0.001, 0.003] |
|  | Cer(d18:1/18:0)/Cer(d18:1/24:0) | 0.003 [0.001, 0.006]* | 0.009 [0.002, 0.016]* | 0.013 [0.005, 0.020] |
|  | Cer(d18:1/18:0)/PC(16:0/22:5) | 0.000 [0.000, 0.001]* | 0.001 [0.000, 0.001]* | 0.001 [0.001, 0.002] |
|  | Cer(d18:1/24:1)/PC(14:0/22:6) | 0.111 [0.029, 0.203]* | 0.219 [-0.036, 0.449] | 0.330 [0.044, 0.594] |
|  | Cer(d18:1/24:1)/PC(16:0/22:5) | 0.002 [0.000, 0.003]* | 0.004 [0.001, 0.008]* | 0.006 [0.003, 0.009] |
|  | CERT1 | 0.398 [0.107, 0.721]* | 0.806 [-0.085, 1.615] | 1.205 [0.195, 2.101] |
|  | CERT2 | 0.295 [0.086, 0.521]* | 0.758 [0.008, 1.486]* | 1.053 [0.264, 1.823] |
| Periodontitis stage | Cer(d18:1/16:0)/Cer(d18:1/24:0) | 0.003 [0.000, 0.009] | 0.017 [0.002, 0.030]* | 0.020 [0.005, 0.034] |
|  | Cer(d18:1/16:0)/PC(14:0/22:6) | 0.030 [0.000, 0.065]* | 0.088 [0.021, 0.160]* | 0.118 [0.044, 0.195] |
|  | Cer(d18:1/16:0)/PC(16:0/22:5) | 0.000 [0.000, 0.001]* | 0.001 [0.001, 0.002]* | 0.002 [0.001, 0.003] |
|  | Cer(d18:1/18:0)/Cer(d18:1/24:0) | 0.003 [0.000, 0.007]* | 0.009 [0.001, 0.016]* | 0.012 [0.003, 0.020] |
|  | Cer(d18:1/18:0)/PC(16:0/22:5) | 0.000 [0.000, 0.001]* | 0.001 [0.000, 0.001]* | 0.001 [0.000, 0.001] |
|  | Cer(d18:1/24:1)/PC(14:0/22:6) | 0.107 [0.000, 0.225] | 0.275 [-0.002, 0.554] | 0.383 [0.093, 0.674] |
|  | Cer(d18:1/24:1)/PC(16:0/22:5) | 0.001 [0.000, 0.003]* | 0.005 [0.002, 0.008]* | 0.006 [0.003, 0.010] |
|  | CERT1 | 0.391 [0.029, 0.787]* | 0.521 [-0.494, 1.394] | 0.912 [-0.223, 1.902] |
|  | CERT2 | 0.287 [0.016, 0.559]* | 0.829 [0.195, 1.542]* | 1.116 [0.401, 1.904] |

**ACME_CI** : **average causal mediation effect** (indirect effect through the mediator “GlycA”), with 95% CI. Significant effects are marked with *, **ADE_CI** : **average direct effect** (effect of exposure on outcome not through the mediator), with 95% CI. Significant effects are marked with *. **Total_CI**: The **total effect** (direct + indirect) with 95% CI.

**Table S4.** Estimated ACME (indirect), ADE (direct), and total effects of periodontitis stage, periodontitis grade, and dentition status on lipid outcomes (CERT1 and CERT2) mediated by GlycA. Effect sizes with p-values are shown.

| **Exposure** | **Mediator** | **Outcome** | **ACME** | **ACME_p** | **ADE** | **ADE_p** | **Total** |
| --- | --- | --- | --- | --- | --- | --- | --- |
| periodontitis stage | Glyc A | CERT1 | 0.403 | 0.046 | 0.470 | 0.360 | 0.873 |
| periodontitis stage | Glyc A | CERT2 | 0.292 | 0.064 | 0.624 | 0.142 | 0.917 |
| periodontitis grade | Glyc A | CERT1 | 0.386 | 0.020 | 0.725 | 0.100 | 1.111 |
| periodontitis grade | Glyc A | CERT2 | 0.281 | 0.040 | 0.583 | 0.124 | 0.864 |
| dentition status | Glyc A | CERT1 | 0.942 | 0.000 | 0.659 | 0.270 | 1.601 |
| dentition status | Glyc A | CERT2 | 0.663 | 0.000 | 0.035 | 0.908 | 0.698 |

**Figure S1.** Serum ceramide (Cer) and phosphatidylcholine (PC) concentrations and their ratios according to (a) periodontitis stage (I-IV), (b) grade (A-C) and (c) dentition status

**Figure S2.** Serum ceramide (Cer) and phosphatidylcholine (PC) concentrations and their ratios according to (a) tooth loss category and (b) periodontitis progression rate
